# False-Positive PET Uptake in Left Atrial Appendage Closure Devices Due to Postoperative Inflammatory Response

**DOI:** 10.3390/diagnostics16020200

**Published:** 2026-01-08

**Authors:** Marta Hernández-Meneses, Guillermo Cuervo, Marta Tormo-Ratera, Manuel Castellà, Marta Maristany, José María Tolosana, Eduard Quintana, Carlos Falces, Barbara Vidal, Cristina Garcia-de-la-Mària, María-Alexandra Cañas, Jaime Llopis, Asunción Moreno, José María Miró, Andrés Perissinotti, on behalf of the Hospital Clinic Endocarditis Study Group, Clínic Barcelona Nuclear Medicine Group

**Affiliations:** 1 Infectious Diseases Department, Hospital Clinic, Institut d’Investigacions Biomèdiques August Pi i Sunyer (IDIBAPS), University of Barcelona, 08036 Barcelona, Spain; 2 Centro de Investigación Biomédica en Red de Enfermedades Infecciosas (CIBERINFEC), Instituto de Salud Carlos III (ICSIII), 28029 Madrid, Spain; 3 Nuclear Medicine Department, Hospital Clinic, Institut d’Investigacions Biomèdiques August Pi i Sunyer (IDIBAPS), 08036 Barcelona, Spain; 4 Cardiovascular Surgery Department, Hospital Clinic, Institut d’Investigacions Biomèdiques August Pi i Sunyer (IDIBAPS), University of Barcelona, 08036 Barcelona, Spain; 5 Cardiology Department, Hospital Clinic, Institut d’Investigacions Biomèdiques August Pi i Sunyer (IDIBAPS), University of Barcelona, 08036 Barcelona, Spain; 6 Department of Genetics, Microbiology and Statistics, Faculty of Biology, University of Barcelona, 08007 Barcelona, Spain; 7 Reial Academia de Medicina de Catalunya (RAMC), 08001 Barcelona, Spain; 8 Biomedical Research Networking Center of Bioengineering, Biomaterials and Nanomedicine (CIBER-BBN), Instituto de Salud Carlos III (ICSIII), 28029 Madrid, Spain

**Keywords:** cardiac device infection, FDG-PET, CHO-PET, sterile inflammation, left atrial appendage closure device (LAACD), false positives

## Abstract

**Background:** Positron emission tomography (PET) is a valuable tool in the diagnosis of cardiovascular infections. However, increased radiotracer uptake can also be observed in non-infectious inflammatory processes, leading to potential false positives. This study analyzed the uptake related to left atrial appendage closure devices (LAACD—AtriClip^®^) to determine its association with infectious or inflammatory processes. **Methods:** We retrospectively analyzed 28 PET/CT scans from 20 patients with implanted LAACDs: 24 using ^18^F-fluorodeoxyglucose (FDG) and 4 using ^18^F-Choline (CHO). Clinical, laboratory, and imaging data were reviewed, and PET uptake was measured semi-quantitatively. All patients had at least 12 months of follow-up after PET imaging to assess for evidence of device-related infection. **Results:** Homogeneous PET uptake in the LAACD was observed in 93% (26/28) of the PET studies, regardless of the radiotracer used, clinical indication, or time since implantation. Clinical follow-up and laboratory findings revealed no signs of infection related to the LAACD in any case. SUV ratios did not differ significantly between the three PET indication groups (infection, neoplasia, or other; *p* = 0.46), nor between scans performed in patients with and without other confirmed infections unrelated to the LAACD (*p* = 0.37). **Conclusions**: FDG and CHO uptake in LAACDs appears to be a consistent and reproducible finding, most likely reflecting a sterile inflammatory response postoperative inflammatory uptake rather than true infection. Clear recognition of this uptake pattern is important to prevent misinterpretation and reduce the risk of false-positive PET/CT results in patients evaluated for suspected cardiovascular infections.

## 1. Introduction

^18^F-Fluorodeoxyglucose positron emission tomography/Computed Tomography (FDG-PET) is widely used in the diagnosis of cardiovascular infections, including prosthetic valve endocarditis and other cardiovascular infections related to cardiac implantable electronic devices and vascular grafts [1]. In this scenario, an increased uptake of FDG, a glucose analog, is typically associated with areas of heightened metabolic activity, which are often indicative of infection. However, FDG uptake can also occur in sterile inflammatory conditions such as post-surgical inflammation or foreign body reactions, leading to potential misinterpretations and false positive studies [1,2,3,4,5,6,7,8,9].

Analogously, ^18^F-Choline (CHO) is a different valuable PET tracer for assessing tumors with low glucose metabolism (such as prostate cancer or hepatocellular carcinoma, among others) or parathyroid imaging, as its uptake reflects increased cell membrane biosynthesis in malignancies. However, CHO uptake has analogously been documented in inflammatory conditions [10,11,12,13,14,15,16].

The left atrial appendage closure device (LAACD), commercially known as AtriClip^®^, is implanted during cardiac surgery in patients diagnosed with atrial fibrillation, with the aim of reducing the risk of embolic events. In certain instances, the AtriClip LAACD is employed as a substitute for long-term anticoagulation therapy when such treatment is contraindicated or poses significant risks [17,18]. The device is composed of materials selected for their favorable biocompatibility profiles: titanium, known for its excellent corrosion resistance and low biological reactivity; nitinol, a nickel-titanium alloy valued for its flexibility, shape memory, and durability; and Dacron (polyester), a synthetic fiber that encourages tissue integration and reduces thrombotic potential. While these materials are considered non-reactive, they can still trigger a localized, chronic sterile inflammatory response at the implantation site [17,18,19]. This response may lead to increased uptake of molecular imaging tracers such as FDG and CHO, even in the absence of infection [20,21]. A clear understanding of postoperative uptake patterns is crucial to avoid false-positive interpretation. This study systematically characterizes the FDG and CHO uptake patterns in LAACD for the first time and evaluates their relevance in clinical decision-making. This investigation seeks to systematically characterize, for the first time, the patterns of FDG and CHO uptake in patients with LAACDs, and to assess their relevance in clinical decision-making, with emphasis on distinguishing postoperative inflammatory responses from true infectious processes in PET/CT interpretation.

## 2. Materials and Methods

This observational cohort study was conducted at the Hospital Clinic of Barcelona, a tertiary-care university hospital and national referral center for cardiovascular surgery. We retrospectively reviewed all patients referred to our institution between 2019 and 2024 who underwent surgical implantation of a left atrial appendage closure device (LAACD—AtriClip^®^) and subsequently underwent a PET/CT scan, either performed at our center or externally when imaging was provided to our center in DICOM format.

PET/CT scans have been requested for various clinical indications, including suspected infection, fever of unknown origin, oncological evaluation, autoimmune or inflammatory disease, and metabolic disorders. PET/CT was requested for different clinical indications: (1) suspected infection, (2) oncological staging or restaging, and (3) other conditions. For each patient, we collected demographic and clinical variables, including age, sex, comorbidities, immunosuppression status, indication for PET/CT, date of LAACD implantation, and the interval between surgery and imaging. Laboratory data were also collected, including white blood cell count and C-reactive protein (CRP) levels at the time of imaging. To identify any potential infectious process, we systematically reviewed all available clinical records for signs and symptoms of infection (e.g., fever, localized pain, systemic inflammatory response), microbiological results (e.g., blood cultures, site-specific cultures such as respiratory, valvular, or synovial fluid), and any complementary diagnostic procedures.

The primary endpoint of the study was to assess whether PET tracer uptake at the site of the LAACD was consistently present and independent of the clinical indication for the scan. Additionally, we aimed to determine whether this uptake was associated with confirmed LAACD-related infection. All patients were clinically followed for at least 12 months after imaging to confirm the presence or absence of infection involving the device.

### 2.1. Imaging Protocol

Whole-body PET/CT studies at our institution were acquired 60 min after FDG or CHO injection in a hybrid scanner (Biograph mCT 64S; Siemens Healthineers, Erlangen, Germany ) according to EANM procedure guidelines [22]. DICOM images from all PET/CT studies conducted at other centers were gathered and imported.

Myocardial suppression (dietary preparation, 12 h fasting and endovenous administration of hearine 50 IU/kg) was applied only in FDG studies with suspected endocardial infection, according to our institutional protocol.

All PET images (CT-attenuation corrected and non-corrected) were evaluated and analyzed in Syngo.via (Siemens Healthcarer). PET uptake in the region of the left atrial appendage was assessed visually and semiquantitatively by two nuclear medicine physicians measuring the maximum standardized uptake value (SUVmax) of a volume of interest (VOI) including the totality of the LAACD, avoiding the inclusion of any other surrounding uptake. For reference, blood pool-SUVmean was calculated by setting a 3 cm^3^ spherical VOI at the descending thoracic aorta, as well as liver-SUVmean, placing a 5 cm^3^ spherical VOI in the liver, avoiding the inclusion of any abnormal area. With the aim of overcoming any bias related to each subject’s physiological individual fluctuation of radiotracer distribution and PET equipment, SUV ratios were calculated by dividing the SUVmax of the LAACD by the liver SUVmean (liver-SUVr) and blood pool SUVmean (bp-SUVr).

Given the well-established differences in the physiological hepatic uptake between the two radiotracers—specifically, the higher uptake observed with CHO—liver-SUVr values are not directly comparable between tracers. This limitation, combined with the small number of CHO-PET scans (*n* = 4), led to the decision to analyze liver-SUVr values exclusively for FDG-PET studies.

### 2.2. Data Analysis

The presence or absence of LAACD-related infection was determined through a comprehensive review of medical records, complementary diagnostic tests, and clinical follow-up. Clinical indications for PET/CT scans were categorized into three groups: infection, neoplasia, and other conditions (including metabolic, inflammatory, or autoimmune disorders). PET/CT parameters analyzed included SUVmax, liver-SUVr, and bp-SUVr. The interval between LAACD implantation and PET/CT acquisition was calculated in days. Follow-up data were also collected for a minimum of 12 months after imaging to confirm the clinical course and the presence or absence of device-related infection. In cases with suspected infection, the final adjudication regarding the presence or absence of LAACD-related infection was established by the institutional multidisciplinary cardiovascular infection team, integrating microbiological, imaging, and clinical findings. All data were compiled in a structured and anonymized database in compliance with institutional data protection policies.

### 2.3. Statistical Analysis

We analyzed PET/CT findings at the LAACD site and compared clinical, laboratory, and imaging variables between patients with and without confirmed infections. The “infection” group included all patients with a documented infectious process at the time of imaging, although only five cases involved suspected or confirmed endocarditis. The remaining infections were unrelated to the device and included pneumonia and septic arthritis.

Primary endpoints included SUVmax, liver-SUVr, and bp-SUVr values, as well as systemic inflammatory markers (C-reactive protein levels and leukocyte count) in infection versus non-infection groups, and the time interval between LAACD implantation and PET/CT acquisition.

Quantitative variables were expressed as median and interquartile range (IQR), and categorical variables as counts and percentages. Comparisons between diagnostic groups (infection, neoplasia, and other) were performed using one-way analysis of variance (ANOVA) or the non-parametric Kruskal–Wallis test, depending on data distribution. Associations between continuous variables were assessed using Spearman’s correlation coefficient. Discriminant analysis was performed using Wilks’ Lambda to identify variables that significantly differentiated between clinical diagnostic groups. Statistical significance was set at *p* < 0.05 for all analyses. All statistical procedures were carried out using Stata version 14 (StataCorp LLC, College Station, TX, USA).

## 3. Results

A total of 20 patients with implanted LAACD (AtriClip^®^) devices were included in the study, in whom 28 PET/CT scans were performed (24 using FDG and 4 using CHO). Five of these scans were acquired at external institutions using different PET/CT systems. Two patients underwent one additional follow-up scan, and one patient had a total of seven scans over a three-year period. The device was implanted mainly for atrial fibrillation with contraindication to anticoagulation, or for stroke prevention during cardiac surgery.

**Figure 1:**
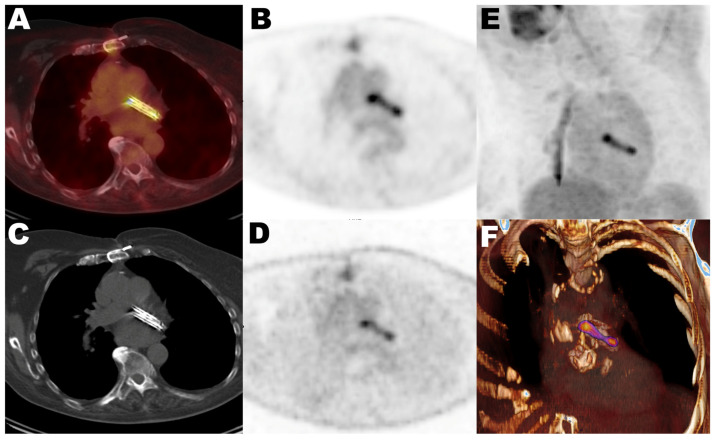
Representative case showing radiotracer uptake in axial images of FDG-PET/CT (**A**), attenuation-correction FDG-PET (**B**), CT (**C**) and non-attenuation-correction FDG-PET (**D**), as well 3D volumetric reconstruction of FDG-PET (**E**) and hybrid FDG-PET/CT (**F**). The most common uptake pattern observed across positive studies was characterized by homogeneous radiotracer activity related to the LAACD, with more prominent activity at the extremities of the device.

**Figure 2:**
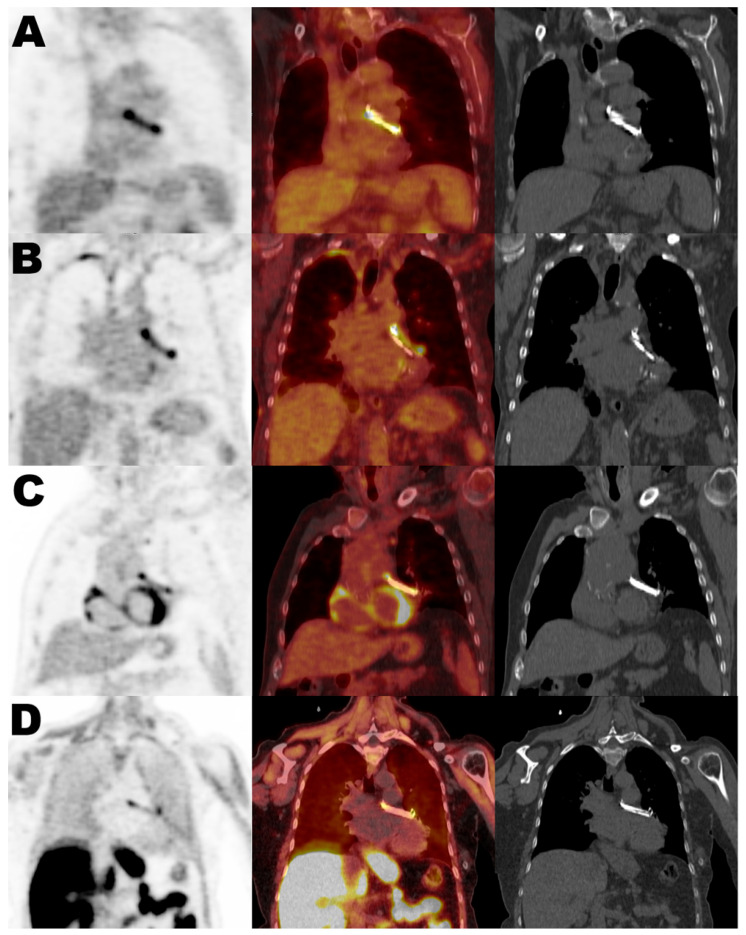
Representative coronal PET, PET/CT and CT images from each group: (**A**) FDG-infection, (**B**) FDg-neoplasia, (**C**) FDG-other conditions and Cho-neoplasia (**D**); demonstrating similar homogeneous LAACD uptake (more prominent activity at the extremities of the device) regardless of clinical indication and radiotracer.

Table 1 summarizes the main demographic, clinical, and imaging characteristics of the study cohort. PET/CT indications were grouped into three clinical categories: infection (8 studies in 7 patients, 35%), neoplasia (16 studies in 9 patients, 45%), and other conditions (4 studies in 4 patients, 20%), including autoimmune, inflammatory, or metabolic disorders. Within the infection group, 5 patients had confirmed endocarditis, and the remaining cases included septic arthritis (*n* = 1) and pneumonia (*n* = 1). All patients in the infection group were receiving antibiotic therapy at the time of PET/CT. The mean duration of antibiotic treatment before imaging was 7 days (range 2–12 days). In all cases, diagnosis was supported by microbiological confirmation through blood, synovial, or respiratory cultures, as applicable. The average time between LAACD implantation and PET/CT imaging was 606 days (range: 70 to 2655 days). All patients were clinically followed for at least 12 months (median follow-up was 40 months) after PET/CT acquisition, and none developed signs or symptoms of LAACD-related infection or elevated inflammatory markers during the follow-up period. Despite the absence of confirmed device-related infection, PET tracer uptake at the LAACD site was observed in 93% (26/28) of the scans, regardless of the radiotracer used, clinical indication, or time since implantation. This finding did not lead to any change in management. Figure 1 shows the most frequent uptake pattern in all positive studies, displaying homogeneous activity related to the LAACD, with more prominent activity at the extremities of the device. This uptake was present in non-attenuated images, and no PET/CT scan showed CT morphological features suggestive of infection. Figure 2 illustrates representative PET/CT images from each of the three clinical groups, demonstrating the similar uptake pattern observed at the LAACD site. Table 2 details quantitative uptake values of all studies specifying the type of tracer, presence/absence of visual LAACD uptake, and clinical indication.

### 3.1. Quantitative Analysis of Tracer Uptake and Inflammatory Markers

#### 3.1.1. Quantitative Differences in Tracer Uptake and Inflammatory Markers by Clinical Group

PET tracer uptake values and systemic inflammatory markers were quantitatively assessed across the three clinical diagnostic groups: infection (*n* = 7), neoplasia (*n* = 9), and other conditions (*n* = 4). As detailed in Table 3, the mean blood pool SUVr (bp-SUVr) was highest in the infection group (4.63 ± 1.68), followed by the neoplasia group (4.38 ± 1.89), and lowest in the group with other conditions (3.43 ± 2.19). However, these differences did not reach statistical significance (ANOVA *p* = 0.46). A similar trend was observed for liver-SUVr of FDG-PET studies, which was numerically elevated in the infection group (3.63 ± 1.24) compared to neoplasia (2.58 ± 1.62) and other conditions (2.18 ± 1.92), with no statistically significant differences (*p* = 0.39). In contrast, C-reactive protein (CRP) levels showed a marked difference between groups. As expected, the infection group had significantly higher CRP values (15.29 ± 4.11 mg/L), in comparison with the neoplasia group (4.86 ± 1.56 mg/L) and the other conditions group (4.43 ± 3.21 mg/L), with statistical significance (ANOVA *p* < 0.01).

#### 3.1.2. Evaluation of SUVr and CRP as Diagnostic Discriminators for Infection Versus Non-Infection

To investigate whether imaging and laboratory variables could discriminate between clinical contexts, a discriminant analysis was performed, including bp-SUVr, liver-SUVr, and CRP. As shown in Table 4, bp-SUVr and CRP were identified as the strongest discriminators among the three clinical groups. The infection group presented a higher bp-SUVr (4.63 ± 1.68) compared to neoplasia (4.38 ± 1.89) and other conditions (3.43 ± 2.19), while CRP levels were distinctly elevated in infection (15.29 ± 4.11 mg/L) vs. neoplasia (4.86 ± 1.56 mg/L) and other (4.43 ± 3.21 mg/L). The overall model showed strong discriminative capacity (F = 52.4, *p* < 0.001). In a separate analysis comparing infection vs. non-infection contexts, liver-SUVr values from FDG-only scans were significantly higher in patients with infection (3.63 ± 1.24) compared to those without infection (2.48 ± 1.68 for neoplasia, 2.18 ± 1.92 for others). This variable demonstrated the highest individual discriminative power (F = 83.8, *p* < 0.001), followed closely by CRP.

**Figure 3:**
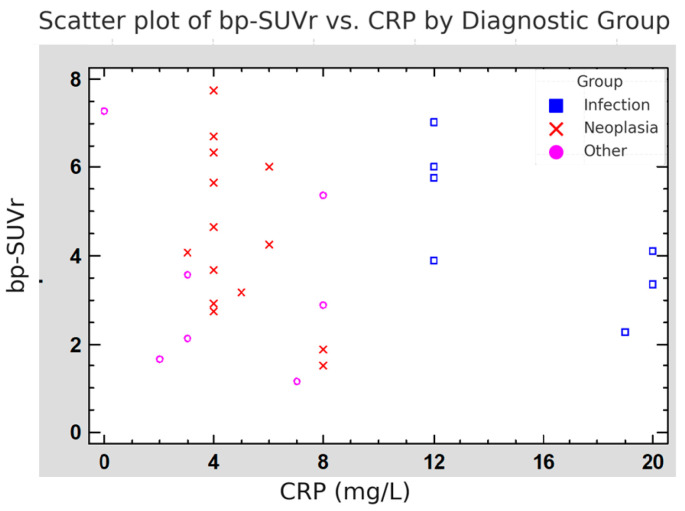
Scatter plot of LAACD bp-SUVr versus CRP level by clinical diagnostic group. Each dot represents one PET/CT study, with groups color-coded as: infection (blue squares), neoplasia (red crosses), and other conditions (pink circles). Elevated bp-SUVr is observed even at low CRP levels, particularly in non-infectious cases, supporting the hypothesis of sterile inflammatory uptake.

Figure 3 shows a scatter plot of bp-SUVr versus CRP levels, with each PET/CT study labeled by clinical group. The plot demonstrates that high bp-SUVr values were found even in patients without infection and with low CRP, especially in the neoplasia and other groups. These findings support that tracer uptake at the LAACD site is often present without signs of systemic inflammation, suggesting a non-infectious or sterile inflammatory cause.

## 4. Discussion

This study underscores the consistent FDG -and also CHO- uptake observed in LAACDs, regardless of clinical indication or confirmed infection. Uptake at the device site was present in 93% of PET/CT scans, with no subsequent signs of device-related infection. While FDG-PET is a highly sensitive modality for detecting cardiovascular infections, its specificity in distinguishing infectious from sterile inflammation remains limited. This phenomenon has also been described in other cardiovascular biomaterials, including vascular grafts [5], surgical adhesives like BioGlue [2,23], and some bioprosthetic valves [1]. These materials, despite being biocompatible, can provoke chronic sterile inflammation detectable by FDG-PET. Our results suggest that a similar mechanism is likely responsible for the uptake observed in LAACDs, possibly related to the Dacron (polyester) fabric that surrounds the device. The presence of uptake in CHO-PET studies in our series further reinforces the idea of a shared inflammatory response, independent of the radiotracer used. A thorough understanding of the material composition of LAACDs and their interactions with the different PET radiotracers is crucial, as these factors may significantly influence radiotracer uptake patterns and potentially lead to misinterpretation of PET/CT findings.

Quantitative analysis confirmed no significant differences in SUVmax, FDG-liver-SUVr, or bp-SUVr between infected and non-infected patients, nor across clinical indication groups. However, as expected, CRP levels were significantly higher in the infection group, and discriminant analysis showed that the combination of CRP and SUVr values could statistically distinguish infection from non-infection. Importantly, elevated SUVr values were also found in patients with low CRP, particularly in oncologic and autoimmune cases. This observation reinforces the concept that PET uptake in LAACDs can occur independently of systemic inflammation and may be misinterpreted as infection if not assessed in a clinical context. Some preclinical studies also support our interpretation. In a rat model, Takemiya et al. showed that while FDG uptake occurred both in infected and sterile subcutaneous implants, a bacteria-specific tracer ([^18^F]fluoro-maltohexaose) accumulated only in true infections. This highlights the limited specificity of FDG and reinforces the hypothesis that LAACD uptake may reflect a sterile inflammatory response to device materials rather than infection [24]. Similar findings have been reported with non-cardiac implants. A recent study demonstrated persistent FDG uptake in polypropylene surgical meshes years after implantation, in the absence of infection or clinical symptoms, further supporting the concept of chronic sterile inflammation associated with biomaterials [25,26] that, in our series, could be detected up to 7 years after LAACD implantation.

Altogether, all these findings suggest that FDG (and likely CHO) uptake at the LAACD site is a frequent, likely sterile phenomenon [1,21,22,23,24]. Reliance solely on PET/CT imaging to diagnose infection may lead to false-positive results and unnecessary interventions. Interpretation should always integrate clinical signs, laboratory markers, and microbiological data. In uncertain cases, additional imaging or follow-up strategies may help clarify the diagnostic picture [23,24,25]. In this context, radiolabeled leukocyte SPECT/CT represents a complementary imaging modality in the evaluation of suspected cardiovascular infections. Although its sensitivity is limited for small lesions and nonpyogenic infections, it offers high specificity for detecting active infection and differentiating infective from inflammatory processes, particularly in cases with equivocal FDG-PET/CT findings. Current evidence supports its role in prosthetic valve endocarditis (sensitivity 64–93%/specificity 88–100%) [26,27,28,29,30], cardiac implantable electronic device infections (sensitivity 60–94%/specificity 88–100%) [31,32,33], ventricular assist devices (sensitivity 71%/specificity 100%) [34] and vascular graft infections (sensitivity 81–100%/specificity 82–100%) [35,36,37,38,39].

The relevance of accurately interpreting PET/CT findings in LAACDs is increasing, as their use continues to grow. Recent guidelines and large clinical trials support the systematic implantation of LAACDs during cardiac surgery for stroke prevention in selected patients [40]. These findings are especially relevant given the growing number of patients receiving LAACDs, as recent clinical trials are expanding their use during cardiac surgery for stroke prevention [17,18]. To date, the diagnosis and management of LAACD infections remain poorly defined, with very few cases reported in the literature [41,42,43,44]. To our knowledge, this is the first case series describing consistent PET uptake in LAACDs in the absence of infection. Notably, only one publication has systematically reviewed the clinical presentation and outcomes of LAACD infections, identifying just 12 reported cases [41]. In that review, PET/CT contributed to the diagnosis in only three instances, and in one case, LAACD uptake was observed despite the absence of microbiological or clinical evidence of infection [42,43,44]. These findings highlight both the rarity of true LAACD infections and the risk of false-positive PET findings, reinforcing the need for cautious clinical interpretation of device-related uptake.

This study has several limitations. First, the retrospective design and the small sample size, including only four CHO-PET scans, limit the strength and generalization of our results. Its retrospective design and small sample size limit the generalizability of the findings. The number of cases was low, and the clinical groups were heterogeneous, particularly in the non-infectious category, which included oncologic, autoimmune, and metabolic conditions. Additionally, none of the infections in our cohort involved the LAACD itself, which prevents assessing device-specific infection uptake patterns. However, this limitation is balanced by the primary objective of the study, which was to characterize PET uptake patterns in LAACDs under real-world clinical conditions and their specificity. Also, a small number of PET/CT scans were acquired on external systems, potentially introducing technical variability. The last important limitation is the absence of histopathological confirmation, which prevents us from defining the uptake as sterile with certainty.

Despite these limitations, this is the first study to systematically assess PET tracer uptake at the LAACD site. The use of both FDG and CHO radiotracers, the inclusion of a wide range of clinical indications, and a uniform follow-up of at least 12 months enhance the external validity and reliability of the findings. The consistent visual and quantitative uptake observed in 93% of scans, combined with detailed statistical analyses, including ANOVA, discriminant analysis, and correlation with inflammatory markers, provides strong evidence for a frequent, likely sterile, inflammatory response associated with the device. These analytical approaches strengthen the interpretation and clinical relevance of the observed imaging patterns.

## 5. Conclusions

Our findings demonstrate that FDG and CHO uptake at the LAACD (AtriClip^®^) site is consistently present, observed in 93% of PET/CT scans, regardless of infection status or clinical indication. Quantitative analyses (SUVmax, bp-SUVr, FDG-liver-SUVr) showed no significant differences between infected and non-infected cases. These results suggest that tracer uptake at the LAACD may often reflect a sterile, non-infectious inflammatory response. Therefore, integration of clinical, laboratory, and imaging data is essential to avoid misdiagnosis and inappropriate clinical decisions.

## Figures and Tables

**Table 1:** Summary of demographic, clinical, and PET imaging parameters by diagnostic group *.

Diagnostic Group	Age (Years) Median (IQR)	% Female	Mean Days from Implant to PET	Radiotracer	Leukocytes (/µL)	CRP (mg/L)	Blood Pool SUVr	LAACD Uptake
Infection (*n* = 7)	74 (68–78)	25.0%	698	FDG 8/COH 0	12,794	13.4	1.46	Positive in 8/8
Neoplasia (*n* = 9)	69 (63–74)	12.0%	106	FDG 15/COH 1	10,356	4.9	1.52	Positive in 10/11
Other (*n* = 4) ^1^	71 (66–75)	67.0%	300	FDG 3/COH 1	9540	4.3	1.56	Positive in 3/4

^1^ Other group includes sarcoidosis (*n* = 1), hyperparathyroidism (*n* = 1), and post-cardiac surgery Dressler’s syndrome (*n* = 2). Infection group includes five patients with endocarditis (three aortic prosthetic, one mitral prosthetic, and one device-related), one with septic arthritis, and one with pneumonia. Neoplasia group includes six patients with solid tumors (lung, colon, breast, pancreas, prostate, and renal) and three with hematologic malignancies (two lymphomas and one chronic leukemia). * Hypertension, diabetes, and chronic kidney disease were the main comorbidities in the cohort, with no differences between diagnostic groups.

**Table 2:** Quantitative PET parameters of all studies specifying type of tracer, presence/absence of visual LAACD uptake, LAACD SUVmax, Liver & Blood pool SUVmean, LiverSUVr, bp-SUVr and clinical indication (infection, oncologic, other).

PET Nº	Radiotracer	LAACD Visual Uptake	LAACD SUVmax	Liver SUVmean	Blood Pool SUVmean	Liver-SUVr	bp-SUVr	Clinical Indication
1	FDG	Yes	3.77	2.05	1.65	1.84	2.28	Infection
2	FDG	Yes	5.32	1.67	1.3	3.19	4.09	Infection
3	FDG	Yes	9.1	1.92	1.58	4.74	5.76	Infection
4	FDG	Yes	5.2	1.66	1.33	3.13	3.91	Infection
5	FDG	Yes	6.67	1.52	1.11	4.39	6.01	Infection
6	FDG	Yes	6.08	2.2	1.82	2.76	3.34	Infection
7	FDG	Yes	9.41	1.75	1.34	5.38	7.02	Infection
8	FDG	Yes	11.18	2.13	1.54	5.25	7.26	Infection
9	FDG	Yes	3.57	2.33	1.91	1.53	1.87	Neoplasia
10	FDG	Yes	3.25	2.68	2.15	1.21	1.51	Neoplasia
11	FDG	Yes	8.04	1.79	1.73	4.49	4.65	Neoplasia
12	FDG	Yes	7.05	2.18	1.92	3.23	3.67	Neoplasia
13	FDG	Yes	8.81	2	1.39	4.41	6.34	Neoplasia
14	FDG	Yes	10.74	2.57	1.9	4.18	5.65	Neoplasia
15	FDG	Yes	4.1	2.1	1.5	1.95	2.73	Neoplasia
16	FDG	Yes	12.1	2.59	1.81	4.67	6.69	Neoplasia
17	FDG	Yes	6.87	2.77	2.34	2.48	2.94	Neoplasia
18	FDG	Yes	3.2	2.2	1.5	1.45	2.13	Neoplasia
19	FDG	Yes	3.46	1.29	1.09	2.68	3.17	Neoplasia
20	FDG	Yes	4.15	1.03	0.69	4.03	6.01	Neoplasia
21	FDG	Yes	9.39	2.15	1.75	4.37	5.37	Other
22	FDG	Yes	6.8	2.97	2.35	2.29	2.89	Other
23	FDG	No	2.4	3	2.1	0.80	1.14	Neoplasia
24	FDG	No	2.58	2.84	1.54	0.91	1.68	Other
25	CHO	Yes	4.81	8.24	1.18	0.58	4.08	Neoplasia
26	CHO	Yes	2.42	6.16	0.57	0.39	4.25	Neoplasia
27	CHO	Yes	3.88	14.07	0.5	0.28	7.76	Neoplasia
28	CHO	Yes	2.78	13.35	0.78	0.21	3.56	Other

**Table 3:** PET tracer uptake (bp-SUVr and liver-SUVr) and inflammatory markers (CRP) by clinical indication (infection, oncologic, other).

Clinical Group	*n*	bp-SUVr (Mean ± SD)	Liver-SUVr (Mean ± SD) ^†^	CRP (mg/L) (Mean ± SD)
Infection	7	4.63 ± 1.68	3.63 ± 1.24	15.29 ± 4.11
Neoplasia	9	4.38 ± 1.89	2.58 ± 1.62	4.86 ± 1.56
Other	4	3.43 ± 2.19	2.18 ± 1.92	4.43 ± 3.21
ANOVA *p*-values	-	0.46	0.39	<0.01

^†^ Liver-SUVr values of 24 FDG-PET studies.

**Table 4:** Discriminant analysis of tracer uptake parameters and CRP by clinical diagnostic group based on 28 PET/CT studies (*n* = 20 patients).

Parameter	Group Comparison	Infection (*n* = 8)	Neoplasia (*n* = 16)	Other (*n* = 4)	*p*-Value
CRP (mg/L)	Infection vs. Neoplasia vs. Other	15.29 ± 4.11	4.86 ± 1.56	4.43 ± 3.21	<0.001 ^†^
Liver-SUVr (24 FDG-PET studies)	Infection vs. Non-infection	3.63 ± 1.24	2.48 ± 1.68	2.18 ± 1.92	<0.001 ^†^
Blood Pool SUVr	Infection vs. Neoplasia vs. Other	4.63 ± 1.68	4.38 ± 1.89	3.43 ± 2.19	<0.001 ^†^

^†^ Discriminant analysis (Wilks’ Lambda), *p*-values derived from one-way ANOVA comparing clinical diagnostic groups.

## Data Availability

The data that support the findings of this study are not publicly available due to ethical and legal restrictions. Access to the data may be granted upon reasonable request and approval by the institutional ethics committee.

## Abbreviations

The following abbreviations are used in this manuscript:

CHO	^18^F-Choline
CRP	C-Reactive Protein
CT	Computed Tomography
DICOM	Digital Imaging and Communications in Medine
EANM	European Association of Nuclear Medine
FDG	^18^F-Fluorodeoxyglucose
LAACD	Left Atrial Appendage Closure Device
Liver-SUVr	Liver Standardized Uptake Value Ratio
PET/CT	Positron Emission Tomography/Computed Tomography
SUV	Standardized Uptake Value
Bp-SUVr	Blood Pool Standardized Uptake Value Ratio
VOI	Volume of Interest

## Appendix A

Members of the Hospital Clínic Cardiovascular Infection Study Group, Hospital Clínic-IDIBAPS, University of Barcelona School of Medicine, Barcelona, Spain: Jose M. Miró, Guillermo Cuervo, Marta Hernández-Meneses, Marta Maristany, Asuncion Moreno (Infectious Diseases Service); Cristina García de la María, Maria Alexandra Cañas-Pacheco, Javier García-González, Filip Krivak (Experimental Endocarditis Laboratory); Manel Almela, Climent Casals, Mateu Espasa, Mariana Fernández-Pittol, Francesc Marco, Jordi Vila (Microbiology Service); Rut Andrea, Oriol de Diego, Carlos Falces, Teresa López, José Ortiz, Ander Regueiro, Carlos Roca, Mercè Roque, Laura Sanchis, Marta Sitges, José M. Tolosana, Bárbara Vidal, Jorge Alcocer, María Ascaso, Manuel Castellá, Daniel Pereda, José L. Pomar, Eduard Quintana, Elena Sandoval (Cardiovascular Institute); Irene Rovira, Guillermina Fita (Anesthesiology Department); Andrés Perissinotti, David Fuster (Nuclear Medicine Service); Jose Ramírez, (Pathology Department); Mercè Brunet (Toxicology Service); Dolors Soy, Montse Tuset (Pharmacy Service); Pedro Castro, Adrian Téllez (Intensive Care Unit); David Nicolás (Home hospitalization service); Xabier Urra (Neurology Service) and Jaume Llopis (Genetics and Biostatistics Department, Faculty of Biology, University of Barcelona).

Members of Clinic Barcelona Nuclear Medicine (CBNM) group: África Muxi, Sergi Vidal-Sicart, Xavier Setoain, Pilar Paredes, Mercé Moragas, Sebastian Casanueva-Eliceiry, Amparo Cobo, Andrés Perissinotti, Katherine Quintero, Francisco Campos, Inmaculada Romero, Alba Garcia-Garrit, Arnau Farré-Melero, Aida Niñerola & David Fuster.
